# Factors associated with the periodicity of *Loa loa* microfilaremia in the Republic of the Congo

**DOI:** 10.1186/s13071-022-05541-y

**Published:** 2022-11-09

**Authors:** Jérémy T. Campillo, Frédéric Louya, Paul Bikita, François Missamou, Sébastien D. S. Pion, Michel Boussinesq, Cédric B. Chesnais

**Affiliations:** 1 Institut de Recherche Pour le Développement (IRD), TransVIHMI, Université de Montpellier, INSERM Unité 1175, Montpellier, France; 2 Programme National de Lutte Contre L’Onchocercose, Direction de l’Épidémiologie et de la Lutte Contre la Maladie, Ministère de la Santé et de la Population, Brazzaville, Republic of Congo

**Keywords:** *Loa loa*, Microfilaremia, Periodicity, Temperature, Republic of the Congo

## Abstract

**Background:**

*Loa loa* microfilariae circulate in the peripheral blood of human hosts following a diurnal periodicity, with maximal microfilaremia levels generally observed between 10:00 am and 3:00 pm. Few studies have assessed factors potentially associated with this periodicity.

**Methods:**

Microfilaremia data were collected repeatedly between 9:00 am and 8:00 pm from 13 individuals in the Republic of the Congo. Using local polynomial regression (LOESS), we determined the best models representing the dynamics of microfilaremia over this period. In a second step, using cosinor models, we evaluated the influence of sex, age, and body temperature on the periodicity of *L. loa* microfilaremia in blood.

**Results:**

All subjects reached their maximum microfilaremia between 10:00 am and 4:00 pm. Individual microfilaremia showed different patterns between individuals, and some clearly showed multiple peaks within a day. LOESS provided a good fit to the observed data. Without adjustment, the maximum microfilarial density was reached around 11:00 am. Adjustment revealed three distinct modes of microfilaremia, occurring around 10:00 am, 1:00 pm, and 4:00 pm. Cosinor models also provided good fit to our data. After adjustment on body temperature, the *L. loa* microfilaremia fluctuation amplitude decreased significantly from 1684.8 to 310.6 microfilariae(mf)/ml and the predicted peak was estimated at 12:02 pm.

**Conclusions:**

We characterized the periodicity of *L. loa* microfilaremia mathematically with two different approaches: cosinor models and LOESS regression. Both models suggest that body temperature plays a role in the variation in microfilaremia within a day. Further studies are needed to identify individual co-factors affecting microfilaremia.

**Graphical Abstract:**

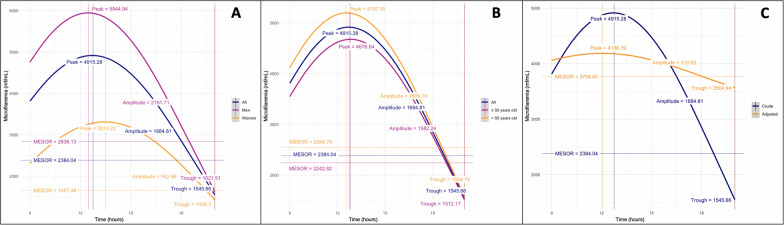

## Background

In 1891, Patrick Manson reported the presence of two new types of microfilariae (mf) in the blood of African patients, “*filaria sanguinis hominis major*” and “*filaria sanguinis hominis minor*,” corresponding to *Loa loa* and *Mansonella perstans*, respectively. He also showed that the former, contrarily to “*Filaria sanguinis hominis*” (*Wuchereria bancrofti*), whose nocturnal periodicity had been described 10 years earlier, circulated in the peripheral blood with a diurnal periodicity [1]: the microfilarial density (MFD) increased in the morning to reach a maximum (often called the “peak”) between 10:00 am and 5:00 pm, and then decreased to reduced levels at night [2]. In 1892, Manson changed the names *Filaria sanguinis hominis*, *F. s. h. major*, and *F. s. h. minor *to *F. s. h. nocturna*, *F. s. h. diurna*, and *F. s. h. perstans* [3]. Natural infection of monkeys with *Loa* sp. was first reported in the 1920s, and Duke and Wijers showed in 1957 that in these hosts, MFD presents a nocturnal periodicity [4]. Hybridization between the human and simian “strains” of *L. loa* has been achieved by experimental infections of drills, and hybrid mf showed a predominantly diurnal periodicity. It is assumed that such hybridization occurs very rarely in natural conditions [5]. As suggested for *W. bancrofti* [6, 7] the time of *L. loa* MFD peak is probably genetically determined and actually corresponds to the time of maximum vector biting activity.

Few studies have analyzed the role of biological factors modulating the periodicity of *L. loa* MFD. In 1921, Low and O’Driscoll studied the effect of reversing the nychthemeral cycle of activity in two subjects infected with *L. loa* (i.e., by asking them to sleep during the day and be active at night) and reported that, contrary to what had been found with *W. bancrofti*, the inverted habits of life had no effect on the diurnal periodicity of *L. loa* MFD [8]. However, the follow-up lasted only 6 days, while similar experiments conducted in subjects infected by *W. bancrofti* showed that complete inversion of periodicity occurred after 10–14 days [9, 10]. In 1950, Kershaw analyzed the periodicity of *L. loa* MFD in nine Cameroonian prisoners living a very regular and early morning life [11]. Although showing wide variations in its amplitude, the course of MFD during the day appeared similar among these nine subjects with an increase and decrease coinciding with dawn and dusk, respectively, and peaks present between 8:00 am and 2:00 pm, with the most frequent occurrence around 9:00 am [11]. In 1955, Hawking collected data from seven hospitalized patients and compared them with those reported by Kershaw. The MFD peaks in the patients ranged between 2:00 pm and 6:00 pm, whereas those found in the prisoners ranged between 8:00 am and 2:00 pm. Hawking concluded that this difference in peak times was probably due to the less regular daytime habits of the patients before their hospitalization (later waking up, later bedtime) than that of the prisoners. In addition, MFD at 10:00 pm in hospitalized subjects was generally between 10 and 20% of their maximum for the day, a much higher proportion than that observed in prisoners at the same time (all < 5%) [12].

In 1967, Hawking et al. hypothesized that body and/or ambient temperature may affect the *L. loa* MFD in the peripheral blood, since inducing a nocturnal rise in body temperature leads to a rapid increase in the latter (the MFD rose to 74% of the daytime MFD peak in an individual whose temperature reached 39 °C after having been placed in a hot bath for 3 h) [13].

In 1976, Ogunba studied the changes in MFD in four adult Nigerians. Samples were taken at 2-h intervals for 14 h. The MFD of two patients showed two peaks (one at 2:00 pm and one at 6:00 pm) while those of the other two patients showed a single peak at 6:00 pm. In three of the four patients, the MFD were higher at 10:00 pm than at 10:00 am. Ogunba hypothesized that this atypical periodicity pattern might be due to a local strain of *L. loa*, whose periodicity would be adapted to the biting behavior of *Mansonia africana,* a mosquito whose biting peak is at 10:00 pm and which allows the development of *L. loa* mf to the third infective larval stage after 12 days [14].

In 1983, Carme studied the periodicity of *L. loa* MFD in five subjects in the Republic of the Congo by taking finger prick blood samples at 6:30 am, 9:00 am, noon, 3:00 pm, and 6:00 pm. His results are in accord with those of the literature, with a peak of MFD at noon and a classical evolution between 6:30 am and 6:00 pm [15].

Few studies have attempted to mathematically characterize the periodicity of microfilaremia over time. In 1997, Simonsen et al. developed an equation relating *W. bancrofti* microfilaremia intensity to the blood sampling time [16]. In 2009, Kamgno et al. mathematically characterized, for the first time, the periodicity of *L. loa* MFD in three groups of Cameroonian microfilaremic individuals: four subjects who had developed a post-ivermectin neurological serious adverse event (SAE), four who had developed a non-neurological SAE, and 14 control individuals. The objective was to assess whether post-ivermectin SAEs could be associated with infection with simian *L. loa*, whose mf show a nocturnal periodicity. Peak MFD were between 10:00 am and 6:00 pm in all the subjects, and thus the hypothesis that simian parasites play a role in the occurrence of SAEs was not confirmed [17].

Thus, so far, it seems that at least three factors are associated with the MFD periodicity pattern in subjects infected with *L. loa*: the individual lifestyle (time of wake-up and bedtime), body temperature and, perhaps, the peak of biting activity of the local vectors. The role of *Mansonia* sp. as vectors needs to be confirmed, but what is sure is that simian *L. loa* is transmitted by *Chrysops* species biting monkeys at dusk [4]. In the present study, we assessed the periodicity of *L. loa* MFD in individuals living in the Lékoumou Department of the Republic of the Congo and evaluated to what extent sex, age, and body temperature were related to the MFD periodicity pattern.

## Methods

### Study population and ethics

The study was conducted in March 2021 in Ouaka, a village located approximately 30 km east of Sibiti, the capital town of the Lékoumou Department. Loiasis is endemic in this region.

This was nested in a trial aimed at evaluating the safety and efficacy of single doses of levamisole in individuals infected with *L. loa*. This trial was approved by the Committee on Ethics in Health Sciences Research (no. 226/MRSIT/IRSSA/CERRSSA) and an Administrative Authorization (no. 469/MSP/CAB/UCPP-19) was released by the Ministry of Health and Population of the Congo [18].

### Procedures

Given the microfilarial periodicity, it had been planned that each participant in the trial would be sampled at the same time of the day at each time point (pre-treatment, and 2, 7, and 30 post-treatment). However, as this regular time of sampling was not assured, it was decided to evaluate whether MFD could be standardized using a formula taking into account the periodicity pattern and the time of sampling. Subjects who had been found microfilaremic during the screening survey but had not been included in the trial proper were invited to participate in this study on periodicity. They received full information on its objectives, and 13 agreed to participate. Each volunteer signed a specific informed consent form and was given compensation to cover the day not worked. The participants stayed in the village health center from 8:00 am to 8:00 pm, without particular physical activity. They were informed that they could leave the study at any time during the follow-up period.

The periodicity of microfilaremia was evaluated between 9:00 am and 8:00 pm. Blood samples were collected from each individual within a 5-min interval around 9:00 am, 10:00 am, 11:00 am, noon, 1:00 pm, 2:00 pm, 3:00 pm, 4:00 pm, 6:00 pm, and 8:00 pm. Temperatures were measured before each blood collection using an infrared thermometer allowing the measurement of forehead and ambient temperature. Blood was drawn by a finger prick with sterile lancets and collected in non-heparinized capillary tubes to prepare calibrated blood smears (50 µl). After dehemoglobinization and Giemsa staining, the smears were examined under a microscope (×100 magnification) to measure the MFD.

### Statistical analyses

The variables of interest were the subjects’ sex and age and the MFD (mf/ml of blood) and the body and ambient temperature (°C) at each point of measurement.

Circular Pearson correlation coefficients between the MFD, the body temperature, and the time of blood collection were calculated and tests for significance were performed. These coefficients allow us to evaluate the correlation between variables that do not follow a linear trajectory over time.

In order to provide the best representation of the MFD fluctuations over time, we firstly used a locally weighted running line smoother (locally estimated scatterplot smoothing, LOESS) regression [19], which is a supervised machine learning approach enabling the generation of a moving average for scatterplot smoothing among the data points. Two models were developed: one with no adjustment and another with adjustment on age, sex, and body temperature. For both models, the same procedure was followed: (i) construction of models with all possible smoothing parameters (between 0.1 and 5), (ii) cross-validation on 30 blocks, and (iii) choice of the best model based on the smoothing parameter decreasing the average error the most.

Subsequently, in order to better understand and evaluate whether there is a periodicity in the changes in microfilaremia, we performed analyses using the cosinor model [20], which enables one to study variables governed by circadian rhythms. Four analyses were done: three unadjusted (on all participants, stratified by sex, and stratified by median age of the subjects) and one adjusted for body temperature in order to highlight the different patterns of MFD changes according to sex and age and to assess the influence of the body temperature on the periodicity. Adjustment consisted in using the standardized residuals from a mixed linear regression fitting MFD according to body temperature, with a random effect at the individual level.

The cosinor model corresponds to the following equation: $y\left( t \right) = M + A\,\cos \phi \,\cos \omega t - \sin \phi \sin \omega t$ where *y* represents the observed MFD and *t* represents the sampling time. The constant *ω* = 2π/24 represents the 24-h periodicity of *L. loa* MFD. The coefficient M represents the 24-h rhythm-adjusted mean defined as the mean value of the MFD. The parameters *A* and *ϕ* represent the amplitude (defined as half of the largest difference in MFD over a 24-h period) and the acrophase (determining the time of peak) of the cosinor model, respectively. A rhythm detection test (also called zero-amplitude test) was used for all cosinor models to test the significance of the estimated model for the population [20].

All analyses were performed using R software (version 3.6.2).

### Results

**Fig. 1:**
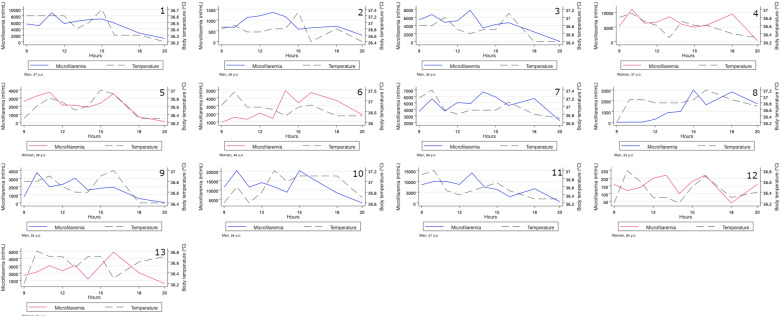
Individual evolution of *Loa loa* microfilaremia and body temperature from 9:00 am to 8:00 pm. Microfilaremia is represented by solid lines (blue for men and pink for women). Body temperature is represented by black dashed lines. *y.o* years

The median age of the participants was 50 years (interquartile range: 37–56) and their mean age was 47.4 years. Of the 13 subjects, eight were male and five were female. Figure 1 represents the individual changes in MFD and body temperature in the 13 subjects. Three had their maximum MFD at 10:00 am, two at 11:00 am, three at 1:00 pm, two at 2:00 pm, one at 3:00 pm, and one at 4:00 pm, and one subject had the same maximum value at 1:00 and 4:00 pm. Individual maximum MFD ranged between 220 (subject 12) and 20,380 mf/ml (subject 10). Figure 1 shows that none of the participants had a unimodal MFD pattern over time.

Circular Pearson correlation coefficients (*r*_circular_) were estimated at −0.05 (*P* = 0.552) between MFD and time of blood sampling, 0.01 (*P* = 0.913) between MFD and body temperature, and 0.29 (*P* < 0.001) between body temperature and time of blood sampling.

**Fig. 2:**
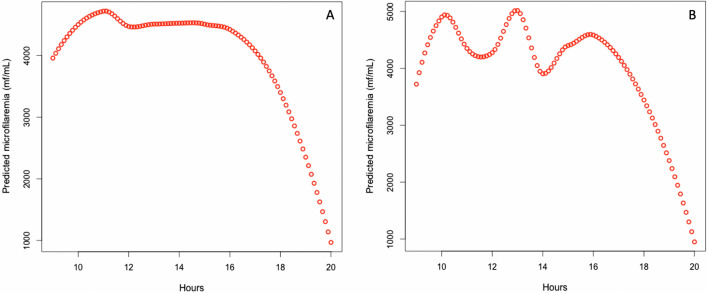
LOESS regression results. (**A**) Unadjusted. (**B**) Adjusted for age, sex, and body temperatures

Figure 2 shows the results of the unadjusted and adjusted LOESS regression on subjects’ age, sex, and body temperature. Without adjustment on temperature, the maximum MFD is reached around 11:00 am. After adjustment, the model reveals three peaks around 10:00 am, 1:00 pm, and 4:00 pm.

**Fig. 3:**
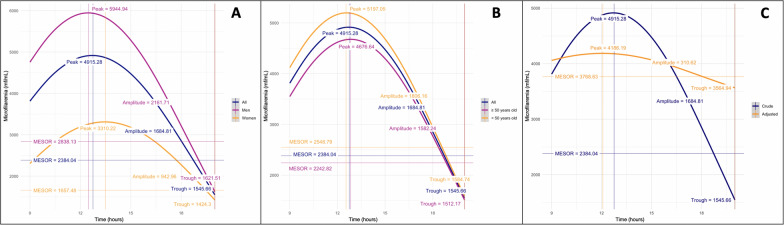
Predicted periodicity of *Loa loa* microfilaremia using cosinor methodology. **A** Model stratified by sex. **B** Model stratified by age (< 50 years and ≥ 50 years). **C** Model adjusted for body temperatures

Figure 3 presents the predicted MFD evolution using the cosinor model between 9:00 am and 8:00 pm for the 13 subjects stratified by sex (panel A), by age category (panel B), and adjusted for body temperature (panel C). The rhythm detection test indicates that the cosinor model fits the data correctly (*P* = 0.013). The model predicts a peak of 4915.3 mf/ml reached at 12:43 pm for the participants taken as a whole. When applied to men only, the peak increased to 5944.9 mf/ml and was reached 15 min earlier, at 12:28 pm. For women, the peak was lower (3310.2 mf/ml) and was reached later, at 1:27 pm. There was little variation between individuals aged less than 50 years and older individuals: a peak of 5197.0 mf/ml reached at 12:35 pm was estimated for the former, and a peak of 4676.6 mf/ml reached at 12:50 pm was estimated for the latter.

After adjustment for body temperature in all 13 subjects, the peak is estimated at 4186 mf/ml, reached at 12:02 pm. The amplitude decreases significantly (from 1684.8 to 310.6 mf/ml). The rhythm detection test is no longer significant (*P* = 0.066), suggesting that body temperature is the major parameter governing periodicity in *L. loa* MFD.

## Discussion

We assessed for the first time the association between age, sex, and body temperature and the periodicity of *L. loa* MFD. We found that the maximum MFD occurred 1 h earlier in male than in female participants, while it was similar between age categories (the difference was only 15 min). After adjusting the models for body temperature, the amplitude of the MFD is strongly decreased (by 81.6%) and the time when microfilaremia is maximum is shifted from 12:43 pm to 12:02 pm. The “experimental” study conducted by Hawking [13] strongly suggested that body temperature induces, either directly or indirectly, an increase in the *L. loa* MFD. Our results confirm this finding. Considering that “there is a lag of approximately 30 min between change of body temperature and change in the microfilaria count,” Hawking hypothesized that “the accumulation or disintegration of some biochemical product (associated with rise of temperature) may be involved” in the decrease in the blockage of the *L. loa* mf in the pulmonary vessels, and thus their release in the general circulation. The “biochemical product(s)” playing a role in *L. loa* microfilarial periodicity still need to be identified. Interestingly, Hawking also found that increasing the body temperature of individuals infected with *W. bancrofti* (either by day or by night) did not induce an increase in the *W. bancrofti* MFD and that increasing the temperature of a mongoose infected with *Monnigofilaria setariosa* and of a *Macaca* monkey infected with *Edesonfilaria malayensis* (both with a nocturnal periodicity) led to a decrease in the MFD [10]. Our results on the time at which the MFD peaks are consistent with the scientific literature and in particular with the 2009 study by Kamgno et al*.* in which MFD peaks were estimated at 12:37 pm, 2:04 pm, and 1:02 pm, depending on their study groups [17].

Nevertheless, individual MFD change over time shows various patterns among individuals, some of which are clearly bi- or trimodal. These patterns could be associated with physiological differences or diverse daytime habits between the individuals or to random variability in the preparation or microscopic examination of the blood smears. Regarding the MFD multimodal pattern, it is interesting to note that in most of the studies on the 24-h human biting activity of *Chrysops* vectors of human *L. loa*, a biphasic pattern, with one peak in the morning and another in the afternoon, is more the rule than the exception [21–26]. Further studies with more detailed data collection, including the lifestyle habits of the individuals, could help understand whether the observed variability in MFD periodicity is random or determined by external or internal factors.

Cosinor models have already been used in parasitology [27], specifically for *Schistosoma mansoni* and *W. bancrofti* [28, 29]. The main advantage of cosinor models is that they can handle non-equidistant data (as is the case here). Cosinor models also have some limitations. They impose the assumption of a normal distribution of the data, which can be a problem in parasitology, where the data often present over-dispersion. In addition, as mentioned above, it is not the best for analyzing multimodal fluctuations within a 24-h period. Furthermore, cosinor models do not allow one to determine the proportion of variability due to external/internal factors or random variability. Another major limitation of the cosinor model is that it considers that every individual has the same periodicity pattern and estimates common parameters. Therefore, it is not possible to determine statistically whether some individuals diverge from the general trend. On the other hand, LOESS regression allows for more accurate modeling of the data through the use of a cross-validation procedure. However, it is possible that such models are subject to overfitting. Compared with cosinor models, the use of this type of model does not assume the normality of the data and allows one to highlight the existence of a multimodal distribution. We found evidence of bi- and even trimodal periodicity after adjusting for body temperature, sex, and age in our study population using this method, which is consistent with the individual observed data.

From a practical point of view, the use of the cosinor, LOESS, or other models could enable one to standardize the MFD of individuals sampled repeatedly in longitudinal studies. As emphasized by Simonsen et al. for *W. bancrofti* [16], the fact that, in such studies, subjects are usually not sampled at exactly the same time of day at the various time points can pose some problems in the interpretation of results. And imposing regular times for the collection of blood of a given individual poses logistical constraints. The results obtained here do not yet allow us to imagine a system allowing the prediction of MFD at a given time from an MFD found at another time in the same subject, but could be a first step toward this goal.

## Conclusions

Although additional studies are needed to determine contributing factors that may impact the periodicity of *L. loa* microfilaremia within a day, our two mathematical approaches (cosinor models and LOESS regression) confirm that body temperature plays a major role in the *L. loa* microfilarial periodicity.

## Data Availability

Data supporting the conclusions of this article are included within the article. The datasets used and/or analyzed during the present study are available from the corresponding author upon reasonable request.
